# The multifaceted role of autophagy in tumor evasion from immune surveillance

**DOI:** 10.18632/oncotarget.7540

**Published:** 2016-02-20

**Authors:** Bassam Janji, Elodie Viry, Etienne Moussay, Jérôme Paggetti, Tsolère Arakelian, Takouhie Mgrditchian, Yosra Messai, Muhammad Zaeem Noman, Kris Van Moer, Meriem Hasmim, Fathia Mami-Chouaib, Guy Berchem, Salem Chouaib

**Affiliations:** ^1^ Laboratory of Experimental Cancer Research, Department of Oncology, Luxembourg Institute of Health, Luxembourg City, Luxembourg; ^2^ UMR 1186, Gustave Roussy Cancer Campus, Villejuif, France; ^3^ Centre Hospitalier de Luxembourg, Department of Hemato-Oncology, Luxembourg City, Luxembourg

**Keywords:** autophagy, hypoxia, tumor microenvironment, tumor immunity, cancer immunotherapy

## Abstract

While autophagy is constitutively executed at basal level in all cells, it is activated in cancer cells in response to various microenvironmental stresses including hypoxia. It is now well established that autophagy can act both as tumor suppressor or tumor promoter. In this regard, several reports indicate that the tumor suppressor function of autophagy is associated with its ability to scavenge damaged oxidative organelles, thereby preventing the accumulation of toxic oxygen radicals and limiting the genome instability. Paradoxically, in developed tumors, autophagy can promote the survival of cancer cells and therefore operates as a cell resistance mechanism. The consensus appears to be that autophagy has a dual role in suppressing tumor initiation and in promoting the survival of established tumors.

This has inspired significant interest in applying anti-autophagy therapies as an entirely new approach to cancer treatment. While much remains to be learned about the regulation and context-dependent biological role of autophagy, it is now well established that modulation of this process could be an attractive approach for the development of novel anticancer therapeutic strategies. In this review, we will summarize recent reports describing how tumor cells, by activating autophagy, manage to resist the immune cell attack. Data described in this review strongly argue that targeting autophagy may represent a conceptual realm for new immunotherapeutic strategies aiming to block the immune escape and therefore providing rational approach to future tumor immunotherapy design.

## INTRODUCTION

As defined by Hanahan and Weinberg, the hallmarks of cancer comprise biological capabilities acquired during the multistep development of human tumors [1]. Tumor avoidance of immunosurveillance [2], allowing tumor cells to escape anticancer immune responses or to actively suppress them [2, 3], has been firmly established as an additional hallmark of cancer.

Several types of immune cells are involved in tumor immune surveillance [4, 5]. Briefly, key cells of the adaptive immune system identifying cancer cells are the Cytotoxic T Lymphocytes (CTL) able to recognize tumor-specific antigens exclusively expressed by tumors [6]. Natural Killer (NK) cells of the innate immune system also play an important role in tumor immune surveillance by mechanisms called “missing-self” and “induced-self” recognitions [7]. While the molecular mechanisms by which CTL and NK cells recognize their target tumor cells are fundamentally different, both types of immune cells kill their target primarily by two major pathways: either through the release of cytotoxic granules containing perforin and granzymes into the cytosol of target cells [8], or through Tumor Necrosis Factor (TNF) super family-dependent killing [9].

There is increasing evidence that many tumor types attempt to evade immune system by disabling components of the immune system that have been dispatched to eliminate them and or by activating multiple overlapping mechanisms to escape fully functional immune system [10-12]. Recently, more subtle mechanisms have been described which comprise the establishment of immunosuppressive microenvironment through the recruitment of immunosuppressive cells including regulatory T cells (Tregs) and myeloid-derived suppressor cells (MDSCs) [13, 14]. Both inflammatory cells can suppress the actions of cytotoxic lymphocytes and thus participate to tumor escape from immune cell attack [15, 16]. In addition, it became clear that the majority of immune effector cells recruited to the tumor site displayed a reduced cytotoxicity toward tumor cells and therefore their anti-tumor functions were largely attenuated not only by the immunosuppressive cells but also in response to several microenvironmental factors. Indeed, hypoxic stress in the tumor microenvironment (TME) is considered so far as one of the major mechanisms responsible for tumor evasion from immune surveillance as the majority of mechanisms suppressing the anti-tumor immune functions directly evolve from the hypoxic TME (reviewed in [17, 18]). Furthermore, immune cells in the hypoxic TME not only fail to exert their anti-tumor functions, but also are co-opted to promote tumor growth [19]. In addition, it has become clear that the immune system not only protects the host against tumor development but also sculpts the immunogenic phenotype of a developing tumor and can favor the emergence of resistant tumor cell variants [20].

It has been recently reported that autophagy is frequently increased in established tumors [21], and high level of autophagy is often found in hypoxic TME. Indeed, several studies support the concept that advanced tumors display an “autophagy addiction” that is required to maintain their energy balance [22, 23]. Patients whose tumors had a high autophagic index were less likely to respond to treatment and had a shorter survival compared with those with a low autophagic index [24]. In this context, autophagy has recently emerged as a new player in regulating the progression of hypoxic solid tumors. Indeed, solid tumors are heterogeneous tissues composed of cancer cells and other tumor-associated cells that constitute the tumor stoma (*i.e.*, immune cells, endothelial cells, fibroblasts). In addition to its role as a self-protective mechanism to maintain energy balance and cell survival of various cell types in the tumor, autophagy has emerged as key process in shaping the interaction between cancer cells and tumor stroma components. Indeed, autophagy may act as an unconventional “delivery system” that affects the composition of the tumor secretome. Such a role determines the tumor progression and defines the outcome of the anticancer immune response [25-27]. Moreover, a growing body of evidence indicates that autophagy activation in immune cells exposed to hypoxic stress in TME, is a key component of the efficient immune response. Constitutive autophagy induction in mast cells is involved in the degranulation process, and therefore may impact the recruitment of innate and adaptive immune effectors [28]. Autophagy plays also a key role in macrophage homeostasis, monocytes recruitment and their differentiation into macrophages [29]. Furthermore, autophagy process is involved in antigens presentation by professional antigen-presenting cells (*e.g.,* dendritic cells, B cells), thus promoting effector T cells priming [30, 31]. Interestingly, autophagy contributes to the development, maintenance and survival of T cells [32-34]. In this context, whether the activation of autophagy in hypoxic TME helps or hinders anti-tumor immune response remains to be demonstrated. Understanding the physiological consequences of autophagy in different cell types in the TME is critical when considering therapies that target autophagy. In this review, we summarize recent data describing how autophagy activation under hypoxia confers resistance mechanism to tumor cells and therefore impairs the anti-tumor immune response.

## AUTOPHAGY: A CELLULAR METABOLIC RESPONSE TO STRESS

Autophagy is an evolutionarily conserved catabolic process that allows cells to degrade damaged proteins and their own cytoplasmic material and organelles. These degradation products serve as alternative energy source to maintain cell homeostasis and viability. There are three different forms of autophagy: macroautophagy (referred to hereafter as autophagy), microautophagy, and chaperone-mediated autophagy [35].

Autophagy occurs at basal level in most cells and serves as housekeeping process for clearance of damaged proteins and organelles. Briefly, autophagy is initiated by a nucleation step, mainly dependent on Beclin 1 (BECN1)-VSp15 core complexes, which requires the formation of double membraned structures, called phagophores. During the elongation of the phagophore, which involves several Autophagy-related proteins (ATG), portions of the cytoplasm are engulfed and the microtubule-associated protein 1 light chain 3 (LC3)-I is lipidated to LC3-II. Next, the phagophore is enclosed, maturated upon the action of LC3-II and BECN1 proteins, and leads to the formation of autophagosome. Finally, sequestered materials of the autophagic vacuole are subjected to degradation by lysosomal hydrolases following autophagosome and lysosome fusion [36].

It is now well established that induction of autophagy promotes cell survival as adaptation response to multiple stresses (*e.g*., starvation, hypoxia, unfolded protein response) [37]. In healthy cells, autophagy activation prevents DNA alteration and genomic instability, which may lead to cancer initiation [38-40]. However, in established tumors, the role of autophagy is still controversial [41]. On one hand, autophagy acts as tumor-suppressor by restricting tumor cell necrosis, which is likely to favor tumor-promoting immunity [42]. Moreover, autophagy also restricts cancer cell proliferation by favoring oncogene-induced senescence [43]. On the other hand, since autophagy is a survival mechanism under stress conditions, its induction in tumor cells results in more aggressive phenotype and resistance to anticancer therapies. Such a role is especially observed in solid tumors which are often exposed to hypoxia [44].

## MECHANISMS INVOLVED IN THE ACTIVATION OF AUTOPHAGY UNDER HYPOXIA

**Figure 1:**
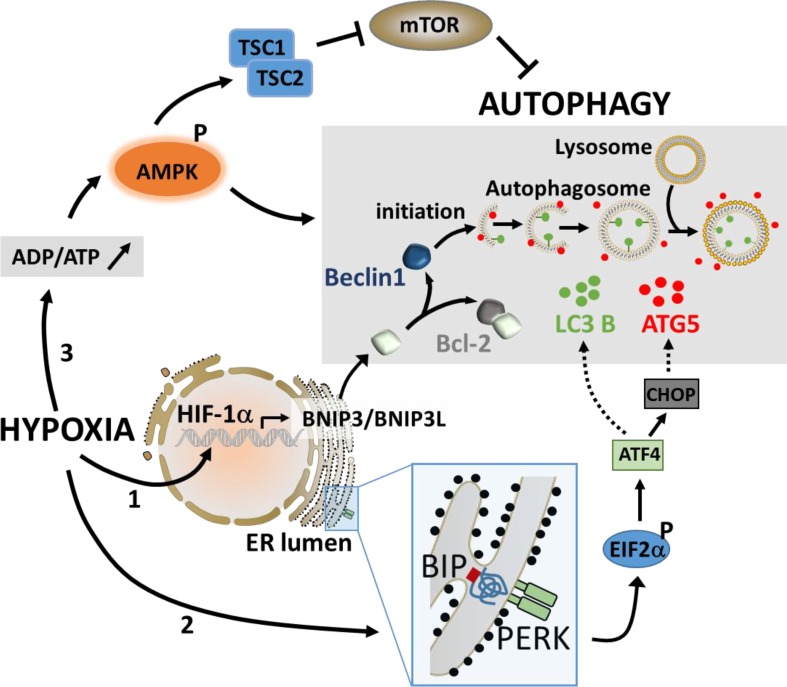
The major pathways involved in the activation of autophagy under hypoxic stress Under hypoxia, the stabilization of HIF-1α leads to its translocation to the nucleus and a rapid induction of the BH3-only proteins (BNIP3 and BNIP3L) through its binding to the hypoxia response element in the promoter of BNIP3. The induction of BNIP3 and BNIP3L displaces Beclin1 from Bcl-2, leading to the induction of autophagy (1). The activation of autophagy by UPR involves PERK which detects unfolded protein. The activation of PERK induces ATF4 *via* phospho-EIF2α and regulates the expression of LC3 and ATG5, two essential components of the autophagy machinery (2). Autophagy can also be induced during hypoxia following an increase in the intracellular ADP/ATP ratio. This leads to the activation of AMPK which subsequently activates autophagy directly or indirectly through the inhibition of mTOR (3).

During hypoxia, autophagy is activated by sensors that detect low oxygen, unfolded proteins, and energy depletion [45]. Therefore, three major pathways have been described to activate autophagy under hypoxic stress (Figure 1).

### Low O2 pressure

Increasing evidence indicates that 50-60% of tumors grow under hypoxic conditions [46], and that enhanced autophagy promotes tumor cell survival [42]. Although hypoxia-induced autophagy mainly depends on hypoxia-inducible factors (HIFs), anoxia-induced autophagy is HIF independent [47, 48]. It has been reported that under hypoxia, HIF-1α-dependent expression of the BH3-only protein Bcl-2/adenovirus E1B 19 kDa-interacting protein 3 (BNIP3) and the related protein, BNIP3L play a key role in autophagy induction [49, 50]. Indeed, these proteins are downstream targets of HIF-1α and are also able to induce mitophagy to manage reactive oxygen species (ROS) production [51]. Although the promoter of BNIP3 contains two hypoxia responsive elements (HREs), HRE1 and HRE2, it has been shown that the induction of BNIP3 occurs through the direct binding of HIF-1α to HRE2 [52]. Mechanistically, Bellot *et al.* showed that induction of BNIP3 and BNIP3L in hypoxic cells disrupts the Beclin1 (BECN1)/B-cell lymphoma 2 (Bcl-2) complex leading to BECN1 release and the subsequent autophagy induction as an adaptive survival response during prolonged hypoxia [53].

### Unfolded Protein Response (UPR)

Due to the high proliferation rate of cancer cells, the capacity of the endoplasmic reticulum (ER) to process proteins is limited and the accumulation of unfolded and misfolded proteins leads to ER stress in cancer cells [54, 55]. Cellular adaptation to ER stress is achieved by the activation of the UPR [56]. It has been reported that ER stress stimulates the assembly of the pre-autophagosomal structures, the formation of autophagosomes, and the transport to the vacuoles in an autophagy (ATG) protein-dependent manner [57]. More recently, in cancer cells, it has been suggested that autophagy may be induced during hypoxia as a result of signals generated by the UPR. Briefly, the Activating Transcription Factor (ATF) 4-activating protein, PKR-Like ER Kinase (PERK) detects unfolded proteins and induces ATF4 to upregulate the expression of the essential autophagy genes LC3 and ATG5 [58-60]. LC3-I is processed to its active form, LC3-II, and trafficked with the ATG5-ATG12-ATG16 complex to the elongating autophagosomes (reviewed in [45]).

### Energy depletion

Autophagy is believed to sustain the energetic needs of the cell during hypoxia by liberating metabolites that can be oxidized to generate ATP [61]. One way that cells sense and adapt to their energetic requirements is through the energy sensor adenosine monophosphate-activated protein kinase (AMPK). Under physiologic conditions, catabolism maintains a high ratio of ATP:ADP. This drives the adenylate kinase reaction in favor of ADP synthesis and, consequently, the cellular AMP:ATP ratio is low and AMPK is inactive. However, if the cell is subjected to a metabolic stress that interferes with ATP synthesis such as hypoxia, the rise in ADP:ATP ratio activates AMPK which subsequently initiates autophagy both directly and indirectly by inhibiting mTOR [62, 63].

Despite a diverse set of signals which can activate autophagy under hypoxia, it is now clearly admitted that the predominant role of autophagy in cancer cells is to confer stress tolerance, which serves to maintain tumor cell survival [42]. However, emerging data demonstrate that autophagy activation not only enables tumor cells to survive stress conditions during cancer development, but also provides them an intrinsic resistance mechanism against anti-tumor immune response [64]. In the following section, we will summarize how autophagy activation confers tumor cell resistance to escape from antigen specific and natural cell-mediated cytotoxicity by regulating key proteins involved in anti-tumor immune responses.

## HYPOXIA-INDUCED AUTOPHAGY CONTROLS THE PHOSPHORYLATION LEVEL OF STAT3 IN TARGET TUMOR CELLS AND IMPAIRS THE CTL-MEDIATED TUMOR CELL KILLING

**Figure 2:**
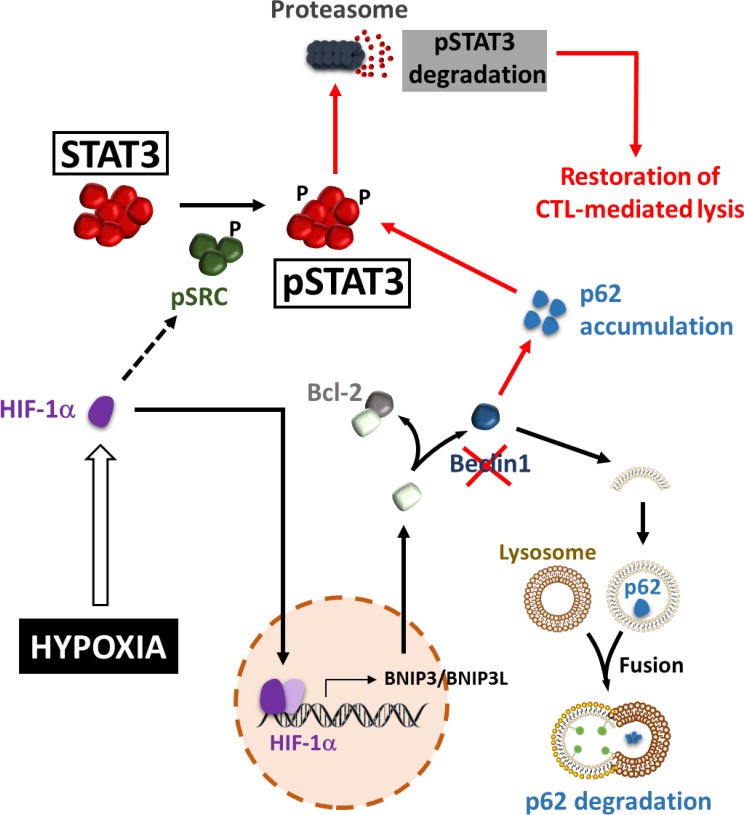
Hypoxia-induced autophagy regulates pSTAT3 and impairs CTL-mediated tumor cell killing Hypoxic stress leads to the accumulation of HIF-1α. By a yet undefined mechanism, HIF-1α increases the level of phospho-Src, which subsequently phosphorylates STAT3 at the Tyr705 residue. BNIP3 and BNIP3L are transcriptionally upregulated by HIF-1α and compete with the BECN1-BCL2 complex. This competition releases BECN1 from the complex and then activates the autophagic machinery. As an autophagy substrate, p62/SQSTM1 is degraded in the autophagosomes following their fusion with lysosomes. When autophagy is blocked (by targeting Beclin1), p62/SQSTM1 is accumulated and promotes the delivery of pSTAT3 to UPS, leading to the acceleration of UPS-dependent degradation of pSTAT3, and the subsequent restoration of CTL-mediated lysis (red arrows).

Several studies have identified Signal Transducer and Activator of Transcription 3 (STAT3) as an important molecule involved in tumor escape from immunosurveillance through the induction of several genes responsible for immunosuppression [65-69]. STAT3 can be activated by many cytokine signaling pathways such as Interleukin (IL)-6 [70]. It is also activated by various growth factor receptor signaling pathways, including Epidermal Growth Factor (EGF) receptor and Vascular Endothelial Growth Factor (VEGF) receptor [71]. Activated STAT3 promotes tumor cell survival, proliferation, angiogenesis/metastasis, and immune escape [72-74]. In the context of immune escape, it has been demonstrated that activated STAT3 can be propagated from tumor cells to several immune cells, mediating a crosstalk between the two cell types, which, in turn, generates immunosuppression of both innate and adaptive immunity [75]. Such propagation is likely mediated by STAT3-regulated factors such as VEGF and IL-10 [68, 69, 76-78]. The first direct evidence for the role of autophagy in the regulation of pSTAT3 was provided by Noman *et al.* who demonstrated that hypoxic lung carcinoma cells can evade CTL-mediated lysis under hypoxia through the induction of pSTAT3 and the activation of autophagy [79, 80] (Figure 2). Indeed, inhibition of autophagy using siRNAs directed against ATG5 or BECN1 restored tumor cells sensibility to CTL-mediated lysis. This was correlated with a decrease in hypoxia-dependent induction of pSTAT3. These results allowed the prediction that blocking autophagy would suppress pSTAT3-dependent survival mechanism making tumor cells more susceptible to CTL attack under hypoxia. Investigating the molecular mechanisms by which autophagy regulates pSTAT3 unraveled that pSTAT3 is selectively degraded by the Ubiquitin Proteasome System (UPS) in autophagy defective tumor cells. This selective degradation involved the adaptor protein Sequestosome1 (SQSTM1/p62). Indeed, this study revealed that the induction of HIF-1α under hypoxia has two effects in tumor cells: i) HIF-1α triggers the phosphorylation of Src, by a yet undefined mechanism, which subsequently phosphorylates the tyrosine residue Y705 of STAT3; ii) HIF-1α activates autophagy by a mechanism involving the increased expression of BNIP3/BNIP3L and the dissociation of the BECN1/Bcl-2 complex. Thus, the activation of autophagy results in the degradation of the p62 protein. When autophagy is blocked, p62 is accumulated and this accelerates the delivery of pSTAT3 to the UPS for selective degradation [81]. These results strongly suggest that autophagy activation by hypoxic stress operates as an intrinsic cell resistance mechanism to prevent immune cell attack. Such a role was also confirmed *in vivo* using B16-F10 syngeneic melanoma model. B16-F10 tumors engrafted in mice are highly hypoxic and autophagy is primarily detected in hypoxic areas of the tumor [80]. Thus, the effect of the autophagy inhibitor Hydroxychloroquine (HCQ) on B16-F10 tumor growth was evaluated alone or in combination with a Tyrosinase-Related Protein-2 (TRP2) peptide-based vaccination strategy. The inhibition of autophagy in B16-F10 engrafted tumors resulted in a significant decrease in tumor growth by inducing apoptosis, as revealed by TUNEL staining. These results strongly argue for a role of autophagy in mediating hypoxia tolerance to the immune system. More interestingly, a significant decrease in tumor growth was observed in vaccinated and HCQ-treated group of mice as compared to control and to treatment alone. Together, these results strongly suggest that *in vivo* inhibition of autophagy improves the anti-tumor effect of a TRP2-based vaccine.

## AUTOPHAGY ACTIVATION IN TUMOR CELLS UNDERGOING EPITHELIAL TO MESENCHYMAL TRANSITION: ROLE IN TUMOR ESCAPE FROM CTL-MEDIATED ANTI-TUMOR IMMUNE RESPONSE

Epithelial to Mesenchymal Transition (EMT) refers to a trans-differentiation program required for tissue morphogenesis during embryonic development [82]. While the role of EMT in cancer cell invasion, metastasis and drug resistance is well established [83, 84], its implication in the regulation of the anti-tumor immune response is only recently reported [85-88].

The relationship between autophagy and EMT in tumors is still not well elucidated so far and studies addressing this issue in the context of tumor immune response are emerging. However, the first evidence for the involvement of autophagy in EMT was described using the epithelial breast adenocarcinoma MCF-7 cell line and its TNF-resistant clone (1001 cells) which has undergone EMT [89]. TNFα was previously described to induce autophagy in MCF-7 cells [90]. Using an autophagy-dedicated microarray, it has been reported that the acquisition of mesenchymal phenotype in 1001 cells was correlated with significant modulation of 47 genes representing 20% of the genes displayed on the autophagy microarray. Among this list, 34 genes were identified as significantly upregulated, while 13 were found significantly repressed in TNFα-resistant mesenchymal 1001 cells compared to TNFα-sensitive epithelial MCF-7 cells. Thus, autophagy gene profiling experiments using autophagy microarray provided strong evidence that EMT is associated with a significant alteration of autophagy gene expression pattern suggesting a concomitant activation of autophagy in mesenchymal cells [91]. This hypothesis was supported by additional results showing the formation of numerous autophagosomes in 1001 compared to MCF-7 cells. The relationship between EMT and autophagy in the context of the anti-tumor immune response was further supported by showing that EMT-induced autophagy represents another mechanism of cancer cell resistance to CTL-mediated lysis [85, 86, 92]. Indeed, activation of the EMT program through the overexpression of Snail homolog 1 (SNAI1) in epithelial cancer cells was correlated with a drastic change in cell morphology and the activation of autophagy flux most likely through the overexpression of BECN1 in mesenchymal cells. Although the precise mechanism by which the EMT affects the expression of BECN1 remained to be addressed, several lines of evidence indicate that this may be related to SNAI1- or EMT-dependent repression of microRNA(s) involved in modulation of BECN1 expression [93, 94]. This result extends the role of EMT as a regulator of autophagy and paves the way to investigate the functional role of EMT-induced autophagy in tumor cells. In this context, it was shown that targeting BECN1 in mesenchymal cells is sufficient to restore CTL-mediated tumor cell lysis, without affecting the mesenchymal morphology and the expression of EMT markers. This finding implies that autophagy is a downstream target of the EMT program in breast cancer cells. Overall, this study suggests that EMT-induced autophagy is a novel mechanism by which tumor cells regulate CTL reactivity and impede their cytotoxic activity, and further points to the complex interplay between the tumor and the immune system.

## HYPOXIA-INDUCED AUTOPHAGY DEGRADES NK-DERIVED GRANZYME B IN TUMOR CELLS AND IMPAIRS TUMOR CELL SUSCEPTIBILITY TO NK-MEDIATED KILLING

**Figure 3:**
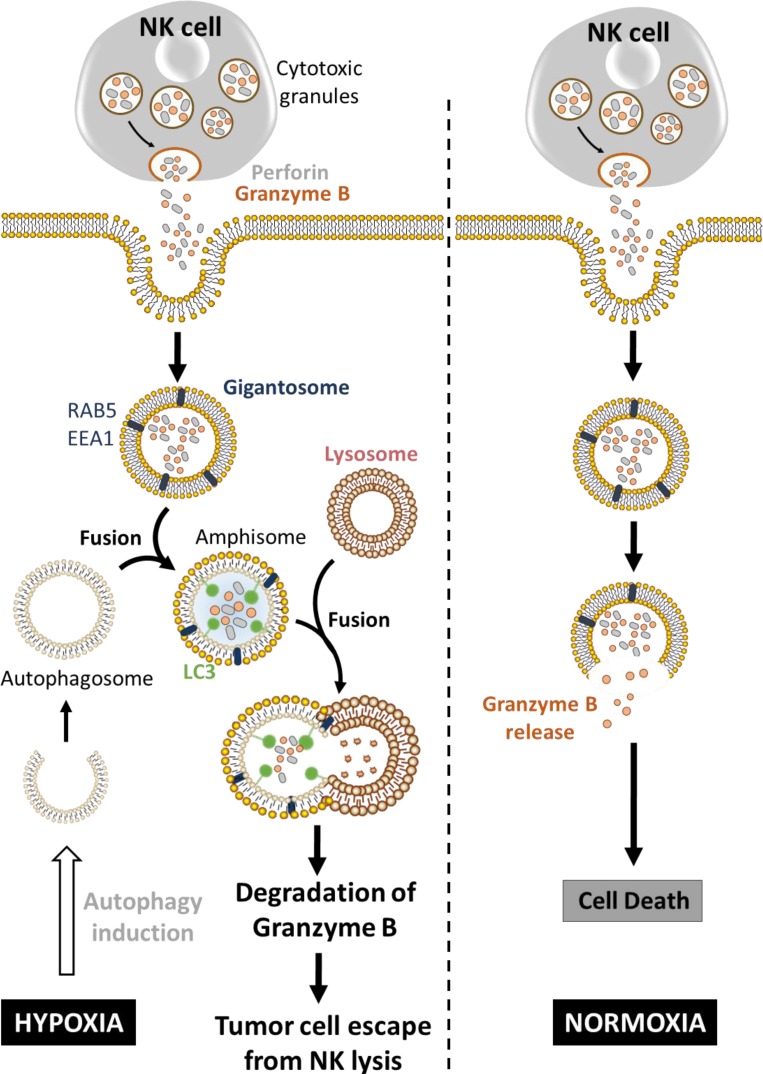
Hypoxia-induced autophagy degrades NK-derived granzyme B and impairs NK-mediated killing Following recognition by NK cells, the cytolytic granules containing perforin and granzyme B enter the target cells through endocytosis and traffic to enlarged endosomes called “gigantosomes” characterized by the expression of endosome markers (RAB5 or EEA1). In normoxic cells, perforin forms pores in the “gigantosome” membrane, allowing granzyme B release and the initiation of cell death. In hypoxic cells, excessive autophagy leads to the fusion of “gigantosomes” with autophagosomes and the subsequent formation of amphisomes containing granzyme B and perforin. The fusion of amphisome with lysosome triggers the selective degradation of granzyme B, making hypoxic tumor cell less sensitive to NK-mediated killing.

Hypoxia-induced autophagy has recently been described as a major player in shaping NK cell-mediated anti-tumor immunity [95]. Evidence has been reported that autophagy is involved in the degradation of NK-derived Granzyme B (GZMB) in hypoxic tumor cells, allowing tumor cell to escape from NK-mediated killing (Figure 3) [96, 97]. Indeed, NK cells recognize and kill their targets by several mechanisms including the release of cytotoxic granules containing perforin and the serine protease GZMB. *In vitro* experimental evidence suggested that both perforin and GZMB secreted by NK cells enter target cells by endocytosis and traffic to enlarged endosomes called “gigantosomes”. Subsequently, perforin forms pores in the “gigantosome” membrane, allowing for the gradual release of GZMB and the initiation of apoptotic cell death [98, 99]. The fusion between early endosomes and autophagic vacuoles to form amphisomes seems to be a prerequisite in some cases for the formation of autolysosomes. Baginska *et al.* described for the first time that GZMB could be exposed to a high risk of being targeted to amphisomes and thereby degraded by autophagy in the lysosomal compartment. Based on these data, an important issue arises from these results: Is GZMB selectively degraded by autophagy or is it just an “innocent victim” which is subject to a bulk nonspecific degradation in hypoxic tumor cells under excessive autophagy? Autophagy has long been considered as a bulk degradation cell process. However, several studies have reported that autophagy can be a highly selective degradation process under stress conditions [100, 101]. The molecular basis of selective autophagy involves several cargo protein receptors such as SQSTM1/p62, Neighbor of BRCA1 gene 1 (NBR1), Optineurin (OPTN) and Calcium-binding and coiled-coil domain-containing protein 2 (CALCOCO2)/NDP52, which are able to interact with ubiquitinated proteins and target them to phagophores for lysosomal degradation [102]. In keeping with this, it is tempting to speculate that GZMB is selectively degraded by autophagy in hypoxic tumor cells. This was supported by several data showing that the level of GZMB in hypoxic cells is restored by targeting the cargo protein p62; and that even if perforin is detected in the same subcellular compartment as GZMB in hypoxic cells, there was no difference in its expression level compared with normoxic cells. While much remains to be learned mechanistically, these data highlight that the degradation of GZMB by autophagy during its intracellular trafficking constitutes a novel mechanism of tumor escape from NK-mediated killing [97]. Furthermore, the role of targeting autophagy in the improvement of NK-mediated anti-tumor immune response *in vivo* was validated using well-characterized melanoma and breast adenocarcinoma syngeneic mouse models. Thus, there was a significant reduction of tumor volume in autophagy-defective melanoma and breast carcinoma most likely as a consequence of potentiation of tumor cell killing by NK cells [96].

## HYPOXIA-INDUCED AUTOPHAGY DESTABILIZES THE IMMUNE SYNAPSE BY CONTROLLING THE EXPRESSION OF GAP-JUNCTIONAL CONNEXIN 43

**Figure 4:**
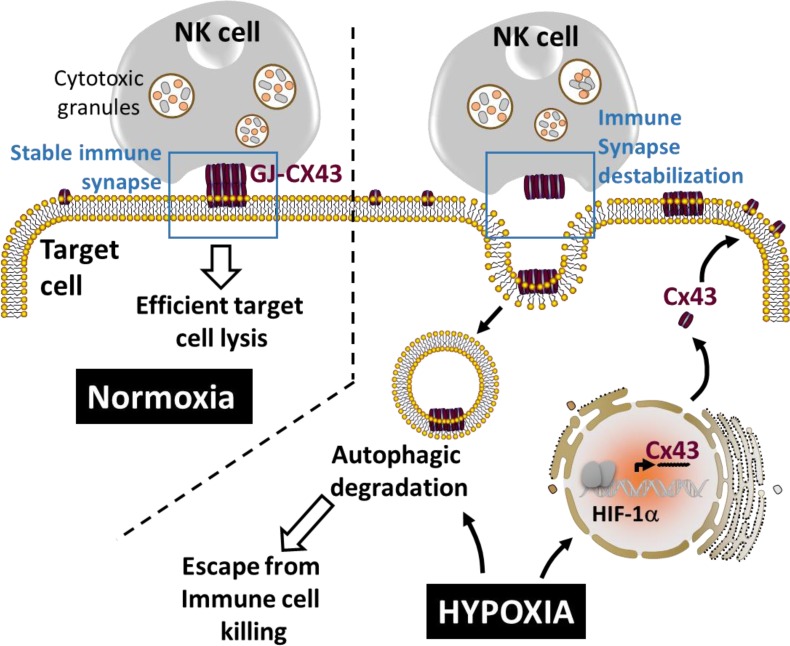
Autophagy destabilizes the immune synapse by controlling the hypoxia-dependent expression of gap-junctional connexin 43 Gap-junctional connexin 43 (GJ-Cx43) is involved in the stabilization of the immune synapse, a prerequisite structure to maintain efficient target cell lysis by NK cells. The overexpression of Cx43 in normoxic target cells improves NK-mediated tumor cell killing. Under hypoxia, HIF-1α induces the transcription of Cx43 which subsequently forms GJ-Cx43 at the immune synapse. Concomitantly, hypoxia-induced autophagy leads to the degradation of GJ-CX43 which subsequently destabilizes the immune synapse and impairs NK-mediated tumor cell killing.

The involvement of autophagy activation in the stability of the immune synapse (IS) between NK and hypoxic melanoma cells has recently emerged [103]. Indeed, it has been shown that hypoxic stress increases the expression of connexin 43 (Cx43), one of the major components of gap junctions [104], in melanoma cells *via* HIF-1α transcriptional activity. Thus, hypoxic melanoma cells displaying increased Cx43 expression were less susceptible to NK-mediated lysis compared to normoxic cells expressing moderate level of Cx43 (Figure 4). Conversely, when overexpressed in normoxic tumor cells, Cx43 improves their susceptibility to NK-mediated killing. This study showed that the immune synapse formed between NK cells and normoxic melanoma cells is more stable and contains higher level of gap-junctional Cx43, whereas the one formed between NK cells and hypoxic cells is less stable and contains significant lower level of gap-junctional Cx43. Moreover, the activation of autophagy in hypoxic melanoma cells selectively degrades gap-junctional Cx43 leading to the destabilization of the IS and the impairment of NK-mediated killing. Inhibition of autophagy by genetic or pharmacological approaches, as well as by expressing an undegradable form of Cx43, significantly restored its accumulation at the IS and improved NK-mediated lysis of hypoxic melanoma cells. This study provides evidence that hypoxic microenvironment negatively affects the immune surveillance of tumors by NK cells through the modulation of Cx43-mediated intercellular communications by autophagy [103].

## HYPOXIA INDUCES THE AUTOPHAGY SENSOR ITPR1 AND IMPAIRS THE NK-MEDIATED ANTI-TUMOR IMMUNE RESPONSE IN CLEAR CELL RENAL CELL CARCINOMA

**Figure 5:**
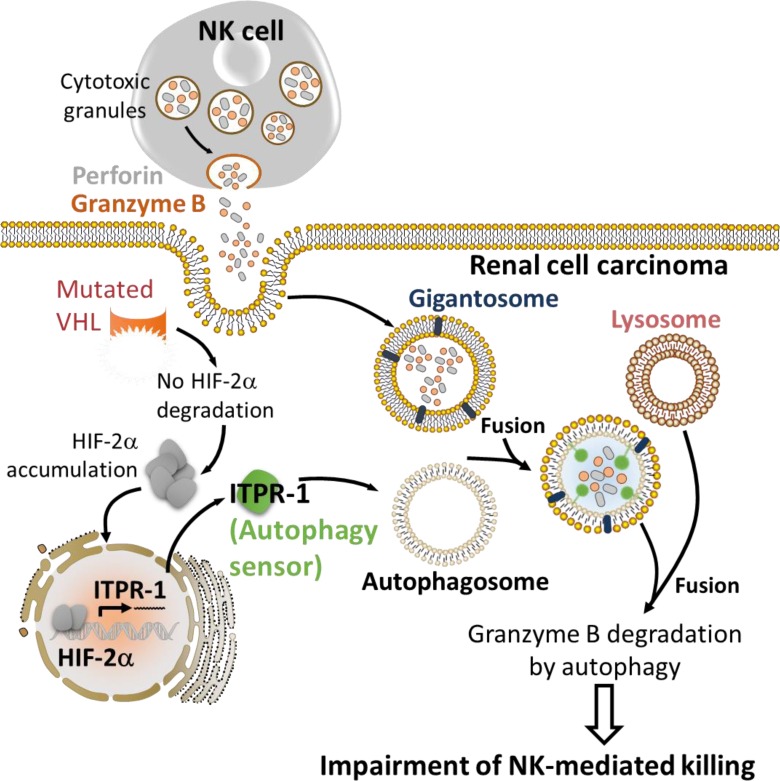
HIF-2α induces the expression of the autophagy sensor ITPR1 leading to the impairment of NK-mediated renal cell carcinoma killing The expression of mutated VHL in renal cell carcinoma leads to the accumulation of HIF-2α. Accumulated HIF-2α translocates to the nucleus and induces the transcription of its target gene ITPR1. ITPR1 plays a key role in sensing a yet undefined signal derived from NK cells to activate autophagy by a mechanism that is not fully understood. The activation of autophagy in renal carcinoma cells expressing the autophagy sensor ITPR1 leads to the degradation of NK-derived granzyme B and ultimately impairs NK-mediated tumor cell killing.

More recently, the role of autophagy in regulating the NK-mediated immune response was extended to other tumor models such as clear cell Renal Cell Carcinoma (ccRCC) [105, 106]. The ccRCC is frequently associated with mutations in the tumor suppressor VHL gene [107]. Such mutations lead to the stabilization and the accumulation of HIF-1α and HIF-2α and their target genes. Using 786-O renal carcinoma cells displaying mutated VHL, it was reported that HIF-2α is stabilized and such stabilization was strikingly associated with the resistance of 786-O cells to NK-mediated lysis since targeting HIF-2α, or decreasing its level by the reconstitution of wild type VHL, restored the resistance of 786-O cells to NK-mediated lysis (Figure 5). These results highlight the critical role of HIF-2α in activating an intrinsic mechanism that makes Renal Cell Carcinoma less sensitive to NK cell attack. Global gene expression profiling identified the inositol 1,4,5-triphosphate receptor, type I (ITPR1) as a new HIF-2α downstream target gene involved in the regulation of NK-mediated anti-tumor immune response. Indeed, ITPR1 was found upregulated in control cells and significantly downregulated in HIF-2α targeted cells. Interestingly, targeting ITPR1 by specific siRNAs significantly restored NK-mediated lysis of these cells. These findings strongly argue that the accumulation of HIF-2α in VHL-mutated 786-O cells leads to the overexpression of ITPR1 which subsequently alters the susceptibility to NK cell attack. Chromatin immuno-precipitation experiment further confirmed that HIF-2α directly induced the expression of ITPR1 by binding to a specific HRE in its proximal promoter. Furthermore, immunochemistry analysis performed in RCC patients, revealed a positive correlation between ITPR1 and HIF-2α expression. The potential involvement of autophagy in the resistance of 786-O cells displaying an overexpression of HIF-1α and ITPR1 was assessed. The results showed that no difference in the basal autophagy level was observed in VHL-mutated and VHL-corrected cells cultured without NK effectors. However, when co-cultured with NK cells, only VHL-mutated 786-O cells were able to activate autophagy. These results imply that the expression of ITPR1 is prerequisite for the induction of autophagy in RCC by a signal derived from NK cells. This was further supported by data showing that siRNA-mediated ITPR1 targeting in 786-O cells abrogates the ability of NK cells to activate autophagy [105, 106]. As discussed in the previous section, the activation of autophagy in target tumor cells impairs NK-mediated tumor cell killing by degrading NK-derived GZMB. In keeping with this, higher level and activity of NK-derived GZMB was detected in VHL-corrected cells as compared to VHL-mutated cells exhibiting increased level of autophagy. Targeting BECN1 in VHL-mutated 786-O cells significantly restored the level and the activity of GZMB. Consistent with the *in vitro* observations, the relevance of HIF-2α/ITPR1/autophagy pathway on NK-dependent anti-tumor immune response using Renca murine RCC was investigated. The results showed a significant decrease in tumor volume in mice engrafted with ITPR1-defective Renca cells as compared to control cells. This decrease is presumably due to the improvement of NK-mediated anti-tumor immune response since the regression of ITPR1 defective tumors was no longer observed in NK-depleted mice. Taken together, these results suggest that inhibiting ITPR1/autophagy in tumors improves their elimination by NK cells *in vivo*. This study highlights that targeting the autophagy sensor ITPR1 could be an alternative strategy to improve NK-mediated anti-tumor immune response in renal carcinoma [105, 106].

It is now clear that HIF-1α and HIF-2α are crucial regulators for the cellular adaptive response to low oxygen tension [108]. However, several studies have also reported that in certain types of cancer, HIF1 and/or -2α are up regulated under normoxic condition. For example, neuroblastoma specimens frequently have strong expression of HIF-2α protein in well-vascularized tumor areas, whereas HIF1α is most restricted to perinecrotic regions [109]. HIF-2α protein can also escape degradation at near normoxic conditions as exemplified by HIF-2α expressing tumor macrophages located close to blood vessels [110]. Recently, stable expression of HIF1α in tumor cells have been found even under normal oxygen tension that mediates immune adaptation through AKT/ERK and VEGFA axis [111]. According to these results, it stands to reason that up-regulation of HIFs, regardless of oxygenation conditions, may act in similar way than under hypoxia. Indeed, it is tempting to speculate that HIFs-mediated induction of autophagy should be also implicated in the development of resistance to immunity even in normoxic tumor areas and/or apparently non-hypoxic early stage cancers. Some evidence has been found in prostate cancer, in which HIF-1α expression is consistently higher in benign prostatic hyperplasia and precancerous tissue compared to normal tissue, and may serve as interesting target for therapy [112].

## AUTOPHAGY BLOCKADE AS THERAPEUTIC STRATEGY FOR IMPROVING ANTICANCER IMMUNOTHERAPY

It is now clearly established that one important consequence of autophagy activation in cancer cells upon stress condition is the development of resistance to radiotherapy [113, 114], chemotherapy [115] and immunotherapy [81, 97].

Several pharmacological inhibitors of autophagy have been identified so far. They can be classified as early- or late-stage inhibitors of the autophagic pathway. 3-Methyladenine (3-MA), wortmannin, and LY294002 targeting the class III PI3K (Vps34) act as an early-stage inhibitors, whereas chloroquine (CQ), hydoxychloroquine (HCQ), bafilomycin A1, and monensin are classified as late-stage inhibitors of autophagy by interfering with lysosomal function. Moreover, microtubule-disrupting agents such as taxanes, nocodazole, colchicine, and vinca alkaloids has been identified as another class of autophagy inhibitors as autophagosomes and lysosomes move along microtubules.

Currently, most of clinical trials registered at National Cancer Institute describe the use of autophagy inhibitors in combination with chemotherapy (http://clinicaltrials.gov). While an increasing amount of preclinical evidence suggest that autophagy induction within the cancer cell alters the antitumor immune response, no clinical data are available so far. However, it stands to reason that several cancer immunotherapeutic strategies based on adoptive transfer of T cells, dendritic cell vaccines, administration of antibodies or recombinant cytokines such as IL-2, could be more effective if the inhibition of the autophagic process is achieved [25, 116]. It has been reported that the combination of high dose of IL-2 with CQ increased long term survival, decreased toxicity associated with vascular leakage, and enhanced immune cell proliferation and infiltration in the liver and spleen of colorectal cancer mice model [117]. This implies that autophagy inhibitors require careful consideration as combinational agents for immunotherapeutic approaches. However, additional preclinical data are needed to understand to which extent and under which circumstances autophagy blockade will improve the therapeutic efficacy of anticancer immunotherapies.

## DISCUSSION

While autophagy has long been considered as an essential mechanism directly involved in several important physiological processes [118], emerging evidence reported in this review highlights that its regulation by microenvironmental factors including hypoxia operates as a cell resistance mechanism for tumor escape from immune surveillance. Such a role of autophagy seems to be related to its ability to regulate multiple proteins and/or factors involved in the anti-tumor immunity.

We believe that cancer cells displaying resistance phenotype following the activation of autophagy are involved in the establishment of tumor supportive microenvironment. Once established, the tumor supportive microenvironment represents a consistently effective barrier to immune cell functions. This is because tumors are not passive targets for host immunity; instead, they actively downregulate the anti-tumor immune responses using different strategies and mechanisms. Such mechanisms include the production by tumor cells of immune modulatory factors and/or an alteration of normal tissue homeostasis occurring in the presence of cancer. The net outcome of these changes is an increased resistance of tumor cells to immune surveillance. In addition, most human tumors appear to be able to interfere with one or more stages of immune cell development, differentiation, migration, cytotoxicity and other effector functions. Thus, all phases of an antitumor immune response are subject to adverse intervention in the tumor microenvironment [19].

In this regard, it is worthy to note that harnessing autophagy for therapeutic purposes requires careful consideration on whether autophagy is induced as a pro-survival mechanism, or is recruited to promote cancer cell killing. Indeed, it is now well established that autophagy plays a critical role in the initiation and progression of tumors. The nature of this role is complex since autophagy can suppress the tumor initiation and reduce genomic instability. Conversely, established tumors appear to utilize autophagy in order to survive periods of metabolic or hypoxic stress. The consensus appears to be that autophagy suppresses tumor initiation, but promotes the survival of established tumors [119]. Consistent with such a complex role it is still difficult to draw a clear conclusion whether, when and how autophagy has to be stimulated or repressed. In addition, the role of autophagy in cancer raises a number of intriguing questions. Does autophagy play a direct or indirect role in cancer development, progression and resistance? If it does, what is its exact contribution? Can autophagy be exploited as a means of enhancing cancer therapies? From the data discussed in this chapter, it appears that, at least, in the context of cancer immunotherapy, strategies aiming to target autophagy could be promising to improve the anti-tumor immune response. However, it is important to highlight that therapeutic strategies targeting autophagy in tumor cells must consider the potential negative impact on immune cells. In addition, most of the studies have focused on the impact of autophagy modulation on tumor cells themselves, but it is more accurate to consider the effect of targeting autophagy in the context of the TME. Indeed, it is now well established that, in the context of tumor immunity, autophagy may influence the cross-talk between cancer and immune cells, leading either to immune-evasion or immune-stimulation. Therefore, a deeper understanding of the impact of autophagy in tumor cells as well as in the TME is necessary to tailor therapies which selectively block suppressive mechanisms and impede anti-tumor response while promoting the anti-tumor immunity. It is our belief that understanding to which extent and under which circumstances inducers and/or inhibitors of autophagy affect the therapeutic efficacy of anticancer treatments will be of great importance to improve the rational use of such modulators.
